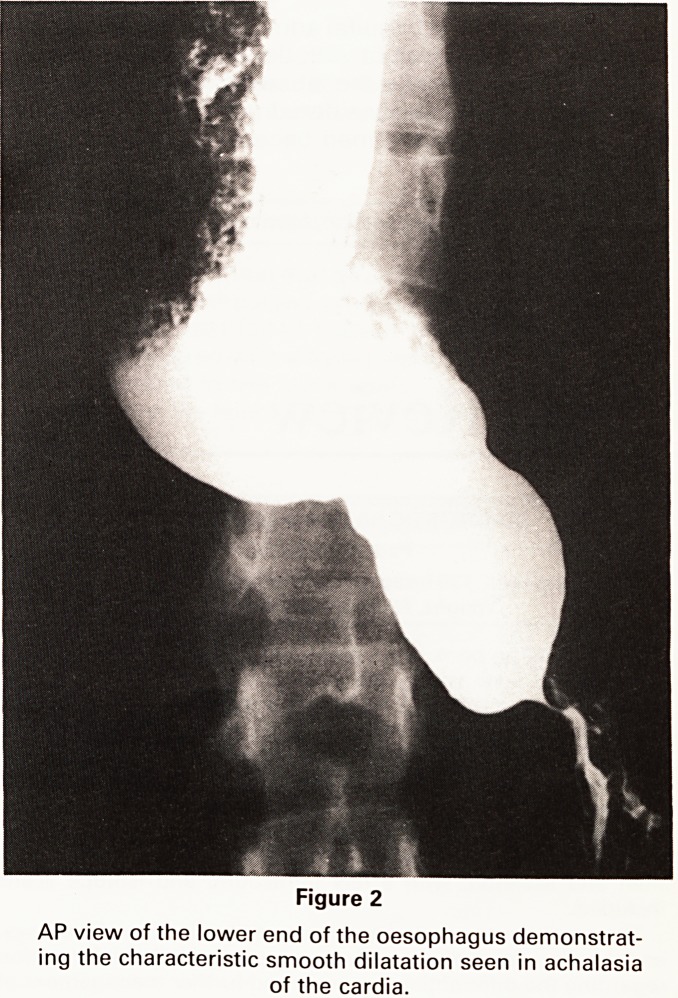# A Case of Dysphagia

**Published:** 1987-11

**Authors:** P. G. P. Stoddart, S. Holl

**Affiliations:** Consultant radiologist, General Hospital, Weston-super-Mare, Avon; General practitioner, Manor Road Surgery, Burnham-on-Sea, Somerset


Bristol Medico-Chirurgical Journal Volume 102 (iv) November 1987

### A case of dysphagia

**P. G. P. Stoddart M. D., F.R.C.R.,**
Consultant radiologist, General Hospital, Weston-super-Mare, Avon.
**S. Holl M.B. B.S.**
General practitioner, Manor Road Surgery, Burnham-on-Sea, Somerset.

### INTRODUCTION

Dysphagia is a common complaint in patients under stress, and in those with an hysterical personality. The simultaneous occurrance of psychiatric and organic disease may make it very difficult to say where organic disability ends and psychogenic overlay begins (Slater and Roth 1969). We present a case of dysphagia in which the diagnosis of underlying organic disease was delayed by the patients' hysterical overlay.

### CASE REPORT

A 25 year old woman was admitted to hospital in the 29th week of her first pregnancy because of dysphagia and severe weight loss. At the time of admission she weighed 49 kilograms, while her normal non-pregnant weight was 50 kilograms. She had consulted her general practitioner for the previous four years complaining of dysphagia for which Valium and Stemetil had been prescribed. She had also had a history of conflict with her in-laws, who had moved to a house close to their son on his marriage to the patient seven years previously; the in-laws became daily visitors, frequently criticising their son. The patient found this interference stressful, and her problems with dysphagia had started at this time.

While in hospital the patient was interviewed by a psychiatrist, who found that she was anxious about the possible effects which her weight loss might have on her child. She complained of difficulty with swallowing, vomiting after meals, and of feeling nervous when eating in front of people. This was confirmed by the ward staff, who found that whenever the patient ate a meal she drew attention to herself, demonstrating that she found it very painful to swallow. A diagnosis of globus hystericus was made; organic obstruction was thought unlikely, although it was recommended that a barium swallow should be obtained following the birth of the child.

A normal child was delivered eleven weeks later, and the patient was discharged home. Her weight fell further to 40 kilograms (height 5 foot 6 inches; normal weight range 55 to 70 kilograms), and she was followed up as a psychiatric outpatient. The diagnosis of anorexia nervosa was made, although it was noted that she had never suffered from amenorrhoea prior to her pregnancy. She slowly regained weight, and was discharged from the clinic four months later, when it was felt that she was poorly motivated.

Six years later the patient moved to a new town, having had two more children in the interim. She went to see her new general practitioner, still complaining of dysphagia. She said that although she had learnt to live

**Figure 1**

PA Chest showing barium and food debris within the oesophagus

**Figure 2**

AP view of the lower end of the oesophagus demonstrating the characteristic smooth dilatation seen in achalasia of the cardia.

with her problem, she was still unable to eat in public. She had found that she could keep solid food down, providing that she drank several glasses of water immediately after each meal. It was discovered that she had never had a barium examination, and this was now requested. The barium swallow demonstrated considerable dilatation of the oesophagus, which had a smooth tapered lower end, absence of peristalsis, and failure of the cardiac sphincter to relax (Figures 1 and 2). Achalsia of the cardia was diagnosed, and the cardiac sphincter was dilated with a Ryder-Mueller balloon. The patient gained 10 kilograms over the subsequent 3 months; she also ate a meal in a public restaurant for the first time in over ten years.

### DISCUSSION

Achalasia of the cardia presents most commonly between the ages of 40 and 70, but it can occur at any age (Carre et al. 1984). Patients typically complain of several years of dysphagia and regurgitation, this leading to avoidance of eating in public (Shearman and Finlayson 1982). The drinking of large volumes of water following eating, in order to prevent regurgitation, is also characteristic, as is the Valsalva manoeuvre to force down solids. In retrospect, 10 years after the patient's initial medical consultation, many of these symptoms of achalasia were present in her history. However dysphagia and regurgitation were the main organic symptoms at the time of the hospital admission. These were exaggerated by the patient's anxiety, and the underlying organic disease was masked by her behaviour; a diagnosis of globus hystericus or anorexia nervosa was a natural conclusion in a young woman with obvious anxiety and known stress at home.

At the time of the hospital admission there were some features which did not fit with the diagnosis of anorexia nervosa (in particular the absence of amenorrhoea). Organic disease was considered possible, but a barium swallow was not performed because of the pregnancy. While pregnancy is a relative contra-indication to X-Ray investigations, there is relatively little danger to a fetus from a barium swallow with modern image intensification, compared to the dangers of severe maternal weight loss. Alternatively an endoscopy could have been performed. In this case the psychiatric diagnosis had become established by the time that the child was born, and the lack of exclusion of organic disease was forgotten.

This case is an extreme example of a common clinical problem: Are symptoms due to organic disease with psychogenic overlay, or is the illness primarily psychiatric? A further problem is that many organic diseases are commonly affected by stress and emotional factors. The problem of differential diagnosis occurs frequently in many areas, including the alimentary tract (e.g. dysphagia, irritable bowel syndrome), the cardiovascular system (e.g. palpitations, cardiac neuroses), the respiratory system (e.g. asthma), and the skin (e.g. pruritus, atopic dermatitis) (Kaplan et al. 1985). The exclusion of organic disease by appropriate investigation is an important first stage in the treatment of psychiatric disease with symptoms which could have an organic basis.

### REFERENCES

CARRE I J, McCRAE WM, MOWAT A P, BRUNT P W, CAMPBELL A G M. Disorders of the Alimentary Tract. In: Forfar J O and Arneil G C, eds. Textbook of Peadiatrics. 3rd ed. (1984). pp 415–524.

KAPLAN H I, KNAPP P H, OKEN D, HALMI K A, HACKETT T P, ROSENBAUM J F, CASSEM N H, WEINER H, ENGELS W D. Psychological factors affecting physical conditions (Psychosomatic disorders). In: Kaplan H I, Sadock B J, eds. Comprehensive Textbook of Psychiatry. 4th ed. Williams and Wilkins, Baltimore (1985) pp 1106–1223.

Shearman D J C, Finlayson N D C. Diseases of the Gastrointestinal Tract and Liver. Churchill Livingstone, London (1982) pp 94–95.

SLATER E, ROTH M. Clinical Psychiatry. 3rd ed. Bailliere, Tindall, and Cassell, London (1969) pp 117.

## Figures and Tables

**Figure 1 f1:**
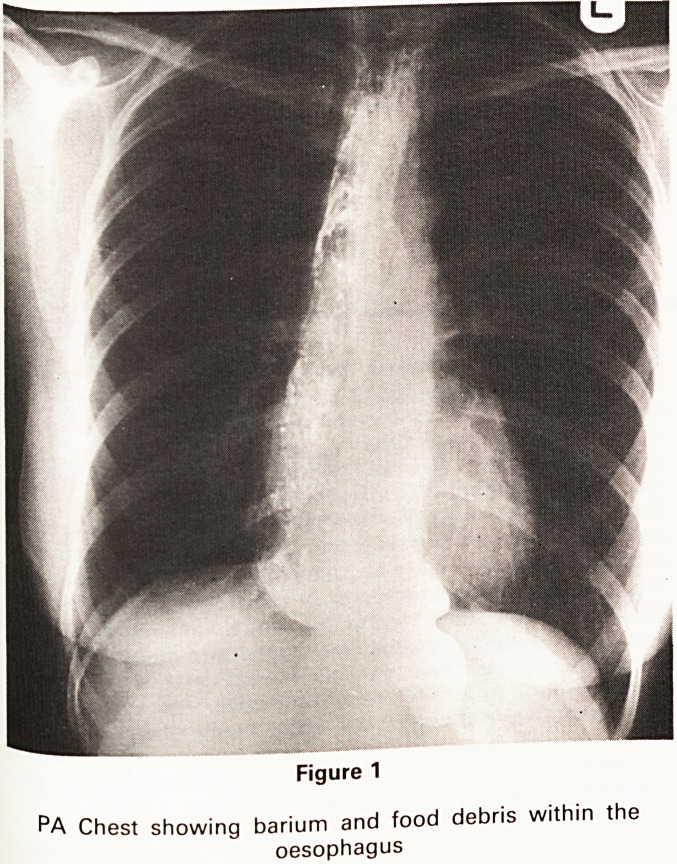


**Figure 2 f2:**